# Risk cognition, agricultural cooperatives training, and farmers' pesticide overuse: Evidence from Shandong Province, China

**DOI:** 10.3389/fpubh.2022.1032862

**Published:** 2022-11-21

**Authors:** Zhong Ren, Haonan Jiang

**Affiliations:** ^1^Business School, Shandong Normal University, Jinan, China; ^2^Warwick Manufacturing Group, University of Warwick, Coventry, United Kingdom

**Keywords:** risk cognition, pesticide overuse, agricultural cooperatives, training, moderating effect

## Abstract

**Introduction:**

Pesticides are widely and excessively used in the world. Reducing pesticide overuse is an important measure to protect the environment and human health.

**Methods:**

Based on the survey data of 518 farmers in Shandong Province, China, using the Logit model to empirically test the effect of risk cognition on farmers' pesticide overuse behavior and the moderating effect of cooperatives training on the effect of risk cognition on farmers' pesticide overuse behavior.

**Results and discussion:**

We found that 21.24% of farmers overused pesticides. The three dimensions of risk cognition have significant negative effects on farmers' behavior of excessive pesticide use, among which the human health risk cognition has the largest impact (0.74), followed by food safety risk cognition (0.68) and ecological environment risk cognition (0.63). Cooperatives training has a positive moderating effect on the relationship between risk cognition and pesticide overuse behavior, that is, when risk cognition matches farmers participating in cooperatives training, the effect on reducing pesticide overuse is more significant. Years of education, planting scale and detection frequency of pesticide residues have significant effects on farmers' pesticide overuse.

**Conclusions:**

The government should help farmers reduce pesticide overuse by improving risk cognition, developing agricultural cooperatives and perfecting guarantee conditions.

## Introduction

The use of pesticides has always been one of the important means to improve land productivity and recover yield loss in agricultural production system (1, 2). However, excessive and inefficient use of pesticides also brings many risks. For example: extensive use of pesticides will directly lead to pesticide residues, and the residual pesticides will accumulate in crops or edible agricultural products, which will bring potential food safety risk (3, 4). Extensive use of pesticides not only causes serious pollution to water, air and soil, but also kills many natural enemies of pests by mistake, destroying the ecological balance of nature (5, 6), resulting in ecological environmental risk. In addition, all pesticides are harmful to human health, and the possible adverse health consequences include dermatitis, neuropsychiatric disorders, cancer, and reproductive function damage (7, 8). In previous surveys in China, Bangladesh, Austria, and other countries and regions, it was found that a certain percentage of farmers had symptoms of discomfort after using pesticides (9–11). Therefore, how to reduce the use of pesticides to reduce the risk of pesticides is a major practical problem to be solved in the sustainable development of agriculture.

Farmers are not only the main body of agricultural production, but also the decision makers of pesticide use. Their pesticide use behavior is the key to reduce the irrational use of pesticides from the source. In order to solve the problem of farmers' excessive use of pesticides, the academia have carried out rich research. Existing studies generally believe that: personal endowments (experience, education level) (12, 13), family endowments (family income, production scale) (14, 15), socio-economic factors (government regulation, social capital, agricultural insurance) (16–18), technical environment (equipment conditions, weather conditions) (19, 20) and others have an important impact on farmers' excessive use of pesticides. It is worth noting that in recent years, some studies have found that farmers' lack of cognitive ability and psychological cognitive level is an important factor for their excessive use of pesticides (21–23). The theory of planned behavior shows that the individual's behavior is the result of deliberate planning, and the individual's cognitive level is the antecedent factor of behavior, which plays an important role in the individual's behavior choice (24). Affected by food safety risk, ecological environment risk and human health risk caused by excessive use of pesticides, farmers' subjective judgment on the above risk uncertainty constitutes their risk cognition (25). Farmers are both the risk cognition subject and the decision-making subject of pesticide use behavior, which conforms to the relevant laws of cognitive behavior theory. Farmers' cognition of the risk of excessive use of pesticides dominates their pesticide use behavior.

However, if farmers can realize the risks of food safety, ecological environment, and human health caused by excessive use of pesticides, will they make necessary behavioral adjustments in the process of using pesticides to reduce the possible risks? Through the investigation of vegetable growers in Iran, Bagheri et al. (26) found that higher cognition of health risk and health costs can help farmers minimize the use of pesticides. A survey by Pan et al. (27) in seven major rice-producing provinces in China found that when farmers have a deep risk cognition, it is easier to reduce pesticide expenditures to reduce possible health risk. However, a survey of farmers in northern Iran by Sharifzadeh et al. (28) found that while farmers' risk cognition is necessary, it is not sufficient to motivate farmers to reduce pesticide use. Jin et al. (17) surveyed in Weifang City, China, and found that almost all farmers can recognize that pesticide overuse may bring risks, but still overuse, frequent and abuse pesticides. It can be seen that the existing literature has not reached a unified conclusion on the relationship between risk cognition and pesticide overuse. The reason may be that the situational factors of different studies are different, that is, there are some external variables that play a moderating role in the relationship between risk cognition and pesticide overuse.

Relevant research shows that there are significant differences between farmers' cognitive level and production behavior in different organizational forms (29). In recent years, in order to realize the organic connection between farmers and modern agricultural development, agricultural cooperatives, and other new agricultural business entities have developed rapidly. Agricultural cooperatives are mutual aid economic organizations that are based on rural household contract management and that are voluntarily united and democratically managed by farmers who produce similar agricultural products (30). Cooperatives can rely on industry, talents, economy, resources and other multiple advantages to carry out training guidance, information exchange and other services (31, 32). Farmers' risk cognition is formed by their continuous acquisition and accumulation of risk information (33), and training is an important channel for them to obtain information (34). Previous studies have confirmed that government training has an important influence on farmers' factor input and technology choice behavior (35, 36), but there are few empirical studies on the relationship between cooperatives training and farmers' pesticide use, the effect of risk cognition on pesticide overuse may also differ among farmers who participate in cooperatives training. Therefore, exploring the relationship between risk cognition and pesticide overuse behavior also needs to focus on the important situational factor of cooperatives training.

As the largest pesticide consumer in the world, China's pesticide consumption far exceeds the economic optimal consumption (37). According to statistics, in 2020, the amount of pesticides used in China was 248,200 tons, the amount of pesticides used per unit area was 3.9 times of the world average, and the utilization rate of pesticides was 40.6%, which was 10–20% points lower than that of developed countries. In order to regulate farmers' pesticide use behavior, the Chinese government has adopted a series of governance measures. In 2015, the Ministry of Agriculture issued the “Implementation Opinions on Fighting the Battle of Agricultural Non-point Source Pollution.” In 2019 and 2020, the “No.1 Document” of the central government put forward the “Action to Promote Pesticide Reduction” for 2 consecutive years. Although the intensity of pesticide use in China has declined at present, China is still the largest pesticide user in the world, and the negative effects caused by farmers' excessive use of pesticides still occur from time to time (38, 39).

**Figure 1:**
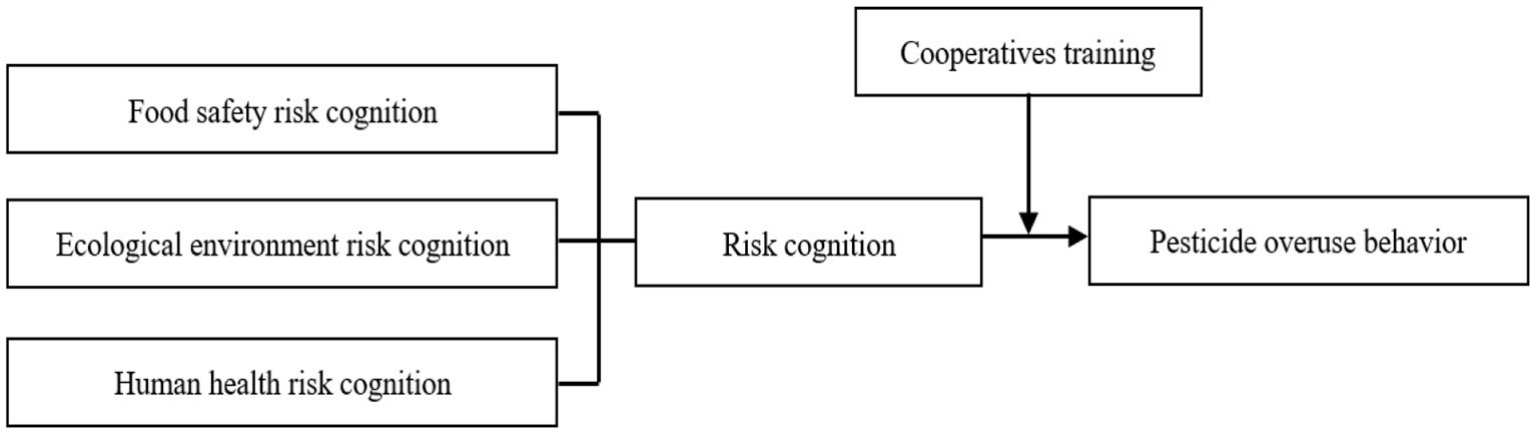
Theoretical analysis framework.

In this study, we use the survey data of farmers in Shandong Province, China, focusing on the relationship between risk cognition, cooperatives training and farmers' pesticide overuse behavior, aiming at solving the following two questions: (1) What is the impact of risk cognition and cooperatives training on farmers' pesticide overuse behavior? (2) Can cooperatives training moderate the relationship between risk cognition and pesticide overuse behavior? These findings will help the government to better understand farmers' pesticide overuse behavior, and provide beneficial enlightenment for formulating pesticide reduction policies. Based on these reasons, this study puts forward the following assumptions (Figure 1):

H_1_: Risk cognition has a significant impact on farmers' pesticide overuse behavior;
H_1a_: Food safety risk cognition has a significant impact on farmers' pesticide overuse behavior;
H_1b_: Ecological environment risk cognition has a significant impact on farmers' pesticide overuse behavior;
H_1c_: Human health risk cognition has a significant impact on farmers' pesticide overuse behavior.
H_2_: Whether farmers participated in cooperatives training moderated the relationship between risk cognition and pesticide overuse behavior;
H_2a_: Whether farmers participated in cooperatives training moderated the relationship between food safety risk cognition and pesticide overuse behavior;
H_2b_: Whether farmers participated in cooperatives training moderated the relationship between ecological environment risk cognition and pesticide overuse behavior;
H_2c_: Whether farmers participated in cooperatives training moderated the relationship between human health risk cognition and pesticide overuse behavior.

The structure of this paper is as follows: Section 1 discusses the relationship between risk cognition, cooperatives training and farmers' pesticide overuse behavior from a theoretical level, and proposes research hypotheses. Section 2 provides an overview of data sources and models. Section 3 presents the findings and discusses. Section 4 summarizes the conclusions and makes policy recommendations.

## Materials and methods

### Data sources

The empirical data comes from our field survey of vegetable growers in Shandong Province, China from September to November, 2021. Shandong Province was chosen because: firstly, Shandong Province is the largest vegetable production and export province in China. Secondly, the situation of pest control in Shandong Province is severe, and the amount of pesticides used ranks first in China. Thirdly, the cooperatives in Shandong Province were the earliest and the largest in China, and the level of development is relatively good.

Before the formal survey, a small-scale preliminary survey was conducted in Jiyang county and Laiwu county of Jinan city. Ten vegetable growers were randomly selected from each county to conduct questionnaire interviews, and the questionnaires were revised and improved. Then, combined with the designation of key vegetable production counties in Shandong Province, the main producing counties were selected by stratified investigation method, and then sample towns were selected from sample counties. Finally, the farmers in villages under the jurisdiction of selected towns were investigated. The survey site includes 40 towns and 120 villages in eight counties of Shouguang, Qingzhou, Shenxian, Laixi, Jiyang, Feixian, Yinan, and Laiwu. According to the suggestion of Krejcie and Morgan (40) on the standard of sample size, we decided to adopt the method of random sampling and distributed 550 questionnaires. Finally, 550 questionnaires were collected, with a recovery rate of 100%, of which 518 were valid questionnaires, with an effective rate of 94.18%. The vegetable varieties in this survey are mainly cabbage, lettuce, and tomato.

The survey mainly uses face-to-face questionnaires to collect data. Interviews were conducted during farmers' leisure time, at home or in other places in the village. Before the interview, we emphasized that participating in this research was optional for farmers, the interview was completely anonymous, and their privacy was well protected. At the same time, we trained eight researchers who participated in the investigation. The questionnaire is in the form of tables and multiple-choice questions, including the basic information of farmers' families, risk cognition, cooperatives training participation and pesticide use behavior. For the respondents who answered seriously and had no contradictory words, the respondents themselves filled out the questionnaire. For those who can't read, researchers will ask questions in the questionnaire to help fill them out. The response time of each questionnaire is about 15 min.

Among the sample farmers, the proportion of males is high, accounting for 87.63% of the total sample. Most of the respondents are between 40 and 60 years old, and rarely receive high school or higher education. Their life experiences are similar. They started farming at about 20 years old, so their farming time is concentrated in 20–40 years, and most of them are in good health. In the family characteristics, the family labor force is mostly 2–3 people, the average planting area is 11.27 mu, the annual household income is generally below 30,000 yuan, and the agricultural income accounts for 78.61% of the total income on average. Generally speaking, the sample distribution is relatively balanced, and it is consistent with the general characteristics of farmers at this stage, which is representative to some extent.

### Variable definition

**Figure 2:**
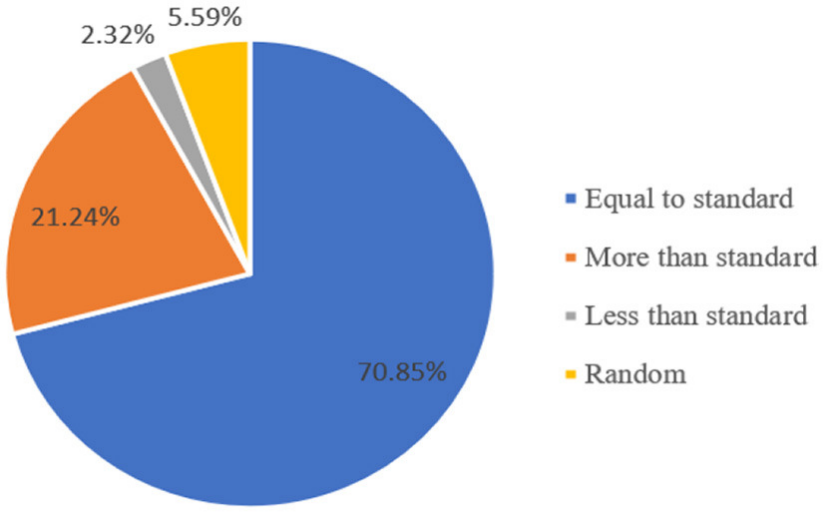
Selection of pesticide use by farmers.

(1) Dependent variable. The indicators of farmers' excessive use of pesticides can be measured from the expenditure of pesticides, the amount of pesticides used and the number of times of pesticides used (9, 27, 41). In view of the differences in the pesticides used by different vegetable varieties, this paper uses whether the farmers' single application exceeds the standard dosage of the pesticide instructions as the criterion for excessive use of pesticides, which is divided into four cases: equal to standard, less than standard, more than standard, and random. The survey results show that 367 households are equal to the standard, accounting for 70.85%; Twelve households are less than the standard, accounting for 2.32%; Twenty-nine households are randomly used, accounting for 5.59%; One hundred and ten households are more than the standard, accounting for 21.24%. It shows that nearly 1/5 farmers in the sample have overused pesticides (Figure 2).

(2) Key variable. The key variable of this paper is risk cognition. According to the general model of cognitive processing, the types of external information that individuals acquire are different, and the risk cognition formed by individuals are also different (42). As mentioned above, risk cognition is further subdivided into food safety risk cognition, ecological environment risk cognition, and human health risk cognition. All items are measured using the Likert 5-level scale.

(3) Moderating variable. The moderating variable in this paper is cooperatives training. Referring to previous studies (43, 44), dummy variables were used to represent whether individuals participated in training to determine training participation behavior. If farmers participate, the value is 1; if farmers do not participate, the value is 0.

(4) Control variables. Existing studies have carried out many explorations on the influencing factors of farmers' excessive use of pesticides, and have roughly formed a common paradigm for analyzing farmers' pesticide use behavior with individual characteristics, management characteristics and external characteristics as logical clues (19, 45, 46). Drawing on the above research results, this paper introduces control variables from the following three aspects: firstly, individual characteristics, including age, years of education, farming experience, and health status. Secondly, management characteristics, including labor endowment, agricultural income proportion, and planting scale. Thirdly, external characteristics, including pesticide residue detection frequency and communication frequency with neighbors.

The variables and statistical characteristics required for empirical analysis in this paper are shown in Table 1.

**Table 1:** Variables and their statistical descriptions.

**Variable**	**Variable meaning and assignment**	**Mean**	**Standard deviation**
Pesticide overuse	Whether it exceeds the standard dosage of pesticide instructions: yes = 1, no = 0.	0.21	0.41
Risk cognition	Pesticide overuse will endanger food safety: Strongly disagree = 1, disagree = 2, generally = 3, agree = 4, strongly agree = 5	2.84	0.76
	Pesticide overuse will destroy the ecological environment: Strongly disagree = 1, disagree = 2, generally = 3, agree = 4, strongly agree = 5	2.78	0.82
	Pesticide overuse will damage to human health: Strongly disagree = 1, disagree = 2, generally = 3, agree = 4, strongly agree = 5	3.06	0.77
Cooperatives training	Whether participated in cooperatives training: yes = 1, no = 0	0.63	0.85
Age	Actual age of respondents/years	48.36	10.12
Years of education	Years of education of respondents/years	8.14	3.08
Farming experience	Years of farming experience of respondents/years	27.92	8.44
Health status	Very poor = 1, poor = 2, fair = 3, good = 4, very good = 5	3.11	0.79
Labor endowment	Number of Household Owned Labor Force in 2021/person	2.48	0.65
Agricultural income proportion	Proportion of agricultural income to total income in 2021/%	78.61	28.85
Planting scale	Planting area in 2021/mu	11.27	41.42
Pesticide residue detection frequency	Local government pesticide residue detection frequency: never = 1, occasionally = 2, often = 3	2.25	0.63
Communication frequency with neighbors	Communication frequency with neighbors: never = 1, occasionally = 2, often = 3	2.48	0.37

### Model construction

(1) Benchmark regression model. The explained variable in this paper is whether farmers have excessive use of pesticides, which is a typical 0–1 variable. Therefore, a binary Logit model is selected for empirical analysis. The specific model settings are as follows:


(1) $$\begin{array}{l} \ln\frac{P_{i}}{1-P_{i}}=\beta_{0}+\beta_{1}X+\beta_{2}Z+\varepsilon \end{array}$$

In the formula, β_0_ is the model intercept; *X* is risk cognition, including food safety risk cognition, ecological environment risk cognition and human health risk cognition; *Z* is the control variables; ε is a random disturbance term; β_1_ and β_2_ are regression coefficients.

(2) Grouping regression. If the influence of the independent variable X on the dependent variable Y changes with the change of the M variable, then the variable M is said to play a moderating role in the relationship between X and Y (47, 48). When X is a continuous variable and M is a categorical variable, grouping regression analysis can be used to test the moderating effect of variable M on the relationship between X and Y. If the difference of the variable X coefficient in the regression results of different groups is significant, it indicates that the variable M has played a significant moderating role in the relationship between X and Y. Since the key independent variable risk cognition in this paper is a continuous variable, and the moderating variable cooperatives training is a categorical variable, a grouping regression model is used to test the moderating effect of cooperatives training in the effect of risk cognition on farmers' excessive use of pesticides.

## Results and discussion

### Multicollinearity test

In order to ensure the validity of the regression results, the multicollinearity among the independent variables was tested first. In this paper, the variance inflation factor (VIF) is used as the indicator of multicollinearity test. Generally, when VIF > 3, it can be considered that there is a certain degree of collinearity among the explanatory variables; when VIF > 10, it can be considered that there is a high degree of collinearity between the explanatory variables. Combining all the test results, the degree of collinearity between the explanatory variables is 1.07–2.83, which are all within a reasonable range, satisfying the principle of independence, and there is no significant collinearity.

### Reliability and validity test

In order to ensure the rationality and validity of the study, this study uses stata16.0 software to analyze and test the reliability and validity of the scale. It can be seen from Table 2 that the Cronbach's α value and CR value of risk cognition, cooperatives training, and excessive pesticide use behavior are all greater than the ideal value of 0.7, which meets the test standard, indicating that the scale has good internal consistency and high reliability. The KMO value of each variable is greater than the ideal value of 0.6, the significant *P*-value of Bartlett's sphericity test is 0.000<0.05, and the KMO value of the overall scale is 0.782, indicating that the scale has good validity. The AVE values of all variables were greater than the ideal value of 0.7, indicating that the scale of this study has good convergent validity. The AVE square root of each variable (Table 3) is larger than the correlation coefficient, which indicates that the research scale has good discrimination validity.

**Table 2:** Reliability and validity test results.

**Variable**	**Cronbach's α**	**CR**	**KMO**	**Bartlett**	**AVE**
Risk cognition	0.879	0.905	0.853	0.000	0.822
Cooperatives training	0.868	0.924	0.841	0.000	0.792
Excessive use of pesticides	0.914	0.928	0.907	0.000	0.746

**Table 3:** Correlation coefficient matrix.

	**1**	**2**	**3**	**4**	**5**	**6**	**7**	**8**	**9**	**10**	**11**	**12**
1	–											
2	−0.213	–										
3	0.661^**^	−0.075	–									
4	−0.416^*^	−0.079	−0.021	–								
5	0.108	0.052	0.009	0.010	–							
6	−0.249	0.311	0.117	0.108	0.082	–						
7	−0.140	0.209	0.101	0.230	0.265^*^	0.377^*^	–					
8	0.021	0.016	0.005	0.005	0.010	0.012	0.243	–				
9	0.114	0.081	0.069	0.188	0.104	0.088	0.020	0.041	–			
10	−0.498	0.438^*^	0.236^*^	0.271	0.353^*^	0.156	0.359^**^	0.331^**^	0.385^**^	(0.907)		
11	0.392	0.307^**^	0.121	0.192	0.289	0.142	0.199	0.077	0.271^*^	0.506^***^	(0.890)	
12	0.265	−0.274^*^	−0.193^*^	0.227	−0.171^*^	0.087^*^	−0.426^**^	−0.419^**^	−0.211^**^	−0.488^***^	−0.396^***^	(0.864)

^***^, ^**^, and ^*^, respectively, represent significant correlation at the level of 1, 5, and 10%. The value in brackets is the square root of AVE. 1 Age; 2 Years of education; 3 Farming experience; 4 Health status; 5 Labor endowment; 6 Proportion of agricultural income; 7 Planting scale; 8 Pesticide residue detection frequency; 9 Frequency of communication with neighbors; 10 Risk cognition; 11 Cooperatives training; 12 Excessive use behavior.

### Discussion of benchmark regression results

Using stata16.0 software to carry out binary Logit regression analysis on farmers' data. Firstly, the control variables were included in the model to obtain model 1; then the risk cognition variables were included into the model to obtain model 2; finally, both risk cognition and control variables were included in the model to obtain model 3 (Table 4). From the regression results, the Pseudo *R*^2^ of Model 3 increased to 0.311, which has stronger explanatory power. Because the sign of regression coefficient can only reflect the influence direction of each variable on farmers' excessive use of pesticides, in order to analyze the influence degree of each variable on farmers' excessive use of pesticides, the marginal effect of each variable is calculated based on the data of Model 3.

**Table 4:** The impact of risk cognition on farmers' excessive use of pesticides.

**Variable name**	**Model 1**		**Model 2**		**Model 3**		**Marginal effect (Based on Model 3)**	
	**Coefficient**	**Standard error**	**Coefficient**	**Standard error**	**Coefficient**	**Standard error**	**Coefficient**	**Standard error**
Food safety risk cognition	–	–	−0.305^**^	0.144	−0.320^**^	0.153	−0.068^**^	0.019
Ecological environment risk cognition	–	–	−0.288^**^	0.121	−0.296^*^	0.127	−0.063^*^	0.016
Human health risk cognition	–	–	−0.319^***^	0.158	−0.345^***^	0.166	−0.074^***^	0.024
Age	0.005	0.006	–	–	0.005	0.006	–	–
Years of education	−0.023^**^	0.033	–	–	−0.026^**^	0.037	−0.006^**^	0.002
Farming experience	0.002	0.012	–	–	0.002	0.013	–	–
Health status	0.009	0.046	–	–	0.011	0.047	–	–
Labor endowment	−0.077	0.093	–	–	−0.084	0.095	–	–
Agricultural income proportion	0.025	0.017	–	–	0.031	0.022	–	–
Planting scale	−0.098^*^	0.063	–	–	−0.101^*^	0.067	−0.022^*^	0.007
Pesticide residue detection frequency	−0.122^**^	0.066	–	–	−0.134^**^	0.070	−0.029^**^	0.012
Communication frequency with neighbors	0.041	0.058	–	–	0.052	0.061	–	–
Prob>chi^2^	0.000		0.000		0.000		–	
Pseudo *R*^2^	0.146		0.235		0.311		–	

Marginal effect analysis only shows significant results;

^*^, ^**^, ^***^, respectively indicate that the significance level of 10, 5, and 1% was passed.

The results of model 3 show that the coefficients of food safety risk cognition, ecological environment risk cognition and human health risk cognition are all significantly negative. It shows that compared with farmers with low cognition of food safety risk, ecological environment risk and human health risk, farmers with high cognition have a lower probability of excessive pesticide use. That is, risk cognition is an important factor affecting farmers' excessive use of pesticides, assuming H_1a_, H_1b_, and H_1c_ are verified. This result was supported by risk aversion theory (49, 50). Farmers, as risk averse people, always make behavior decisions on the basis of predicting the consequences of their behavior choices, and make choices to maximize profits with minimum risks, so as to obtain higher survival guarantee (51). Mohan (52) investigated tea farmers in Nepal and found that the more risk averse farmers are, the more likely they are to obtain agricultural standard certification. Under the limited rational decision-making mode, the more comprehensive farmers' cognition of food safety risk, ecological environment risk, and human health risk caused by excessive use of pesticides, the more inclined they are to use pesticides scientifically and reasonably to avoid risks. The existence of farmers' risk cognition gap leads to the difference of decision-making of excessive pesticide use, so improving farmers' risk cognition is helpful to reduce the probability of excessive pesticide use.

In the research model, the influence of different dimensions of risk cognition on farmers' excessive use of pesticides is asymmetric. The marginal effect results show that the marginal effects of food safety risk cognition, ecological environment risk cognition and human health risk cognition are −6.8, −6.3, and −7.4%, respectively. That is to say, for each unit of increase in food safety risk cognition, ecological environment risk cognition, and human health risk cognition, the probability of farmers' excessive use of pesticides will decrease by 6.8, 6.3, and 7.4%, respectively. It shows that compared with food safety risk and ecological environment risk, farmers are more concerned about the risk of health. This result is similar to the previous research results, which emphasized that farmers' sustainable practices are mainly affected by the factors of maximizing benefits (9, 53). The possible explanation is that human health risk and food safety risk are closer to farmers' own interests, and when farmers feel health damage and food pollution, they are more motivated to reduce the use of pesticides. However, the ecological environmental risk has the attribute of public goods, which will not directly affect farmers' own interests (54). Therefore, as farmers who pursue the maximization of interests, they pay less attention to ecological environmental risk in the decision-making process of pesticide use. However, theoretically speaking, farmers' low cognition of ecological environmental risk may also lead to less impact on excessive pesticide use (27). In our field survey, 76.9% of the sample farmers think that excessive use of pesticides will have an impact on the ecological environment, which is higher than 67.5% of food safety and 74.1% of human health. Even though farmers have the highest level of cognition of ecological environmental risk, the impact of ecological environmental risk on excessive pesticide use is still the smallest. This further proves that the public goods attribute of the ecological environment leads to the minimum impact on the excessive use of pesticides.

Among the control variables, the coefficient of years of education is significantly negative, indicating that farmers with higher years of education are less likely to overuse pesticides. Similarly, Cao et al. (55) and Wang et al. (56) found that farmers with higher education level are more likely to adopt environmentally friendly agricultural practices. The possible reason is that education can improve farmers' knowledge of pesticide use and have more advanced concepts, thereby reducing the possibility of overuse of pesticides. The coefficient of planting scale is significantly negative, indicating that farmers with larger planting scales are less likely to overuse pesticides. This is consistent with the research results of Sharafi et al. (57), which emphasized that there is a positive relationship between business scale and safe application of pesticides. The possible reason is that farmers with larger planting scales have greater demand for pesticides, but in the context of rising pesticide prices, large-scale farmers face greater cost pressure, thereby reducing the possibility of their overuse of pesticides. At the same time, the larger the planting scale, the more dependent farmers are on agricultural production, and they have stronger motivation to take safe production behaviors (42). The coefficient of pesticide residue detection frequency is significantly negative, indicating that farmers are less likely to overuse pesticides in areas with high pesticide residue detection frequency. This is consistent with the findings of Zhao et al. (58), which emphasized that government regulation has a significant impact on farmers' excessive use of pesticides. The possible reason is that the use of pesticides belongs to the “private information” of agricultural producers and operators, and pesticide residue testing can externalize the private information, thereby supervising farmers' pesticide use behavior and reducing the possibility of excessive use of pesticides.

### Moderating effect test of cooperatives training

In order to verify the moderating effect of cooperatives training on the relationship between risk cognition and excessive use of pesticides, group regression was performed according to whether farmers had participated in cooperatives training as the grouping criterion. The estimated results are shown in Table 5. It can be seen that among the farmers who participated in the cooperatives training, the impact of food safety risk cognition, ecological environment risk cognition, and human health risk cognition was significant. Among the farmers who did not participate in the cooperatives training, only the human health risk cognition was significant at the 10% level, and neither the food safety risk cognition nor the ecological environment risk cognition passed the test. This shows that cooperatives training has a moderating effect on the relationship between farmers' risk cognition and excessive pesticide use. This finding is basically consistent with the conclusion that “different organizational forms have a moderating effect between farmers' environmental cognition and environmental behavior” (29). Further research shows that, the effects of the three risk cognition variables in the training group were greater than those in the untrained group. This indicates that when the risk cognition variable is matched with the cooperatives training, the effect of reducing farmers' excessive pesticide use behavior is enhanced, assuming that H_2a_, H_2b_, and H_2c_ are verify. This was supported by the research results of Zhu et al. (59), which pointed out that joining the cooperatives can significantly improve the adoption of green production technology of food and agriculture, with an increase of about 27.16%.

**Table 5:** The moderating effect of cooperatives training.

**Variable name**	**Participated in cooperatives training**		**Have not participated in cooperatives training**	
	**Coefficient**	**Standard error**	**Coefficient**	**Standard error**
Food safety risk cognition	−0.453^***^	0.207	−0.143	0.202
Ecological environment risk cognition	−0.427^**^	0.199	−0.117	0.195
Human health risk cognition	−0.692^***^	0.241	−0.252^*^	0.227
Control variables	Controlled	Controlled
Prob > chi^2^	0.000		0.000	
Pseudo *R*^2^	0.364		0.177	

^*^, ^**^, ^***^, respectively indicate that the significance level of 10, 5, and 1% was passed.

There are several reasons for this: firstly, farmers are generally less literate and have difficulty understanding the risks of pesticide overuse. The cooperatives provides face-to-face, repeated and field technical guidance and training to members through technical service teams, invited agricultural experts, and local agricultural extension personnel (60, 61), enabling farmers to able to fully understand the risk of excessive use of pesticides, have the ability to foresee and monitor errors, and ultimately build a correct cognition system through reflection, evaluation, and error correction, thereby improving farmers' risk cognition level and affecting their pesticide use behavior. Secondly, in the context of small farmers and large markets, the cost of obtaining pesticide quality information is high, and the differentiation of pesticide markets makes it difficult for farmers to use pesticides correctly through experience accumulation (13, 62). Cooperatives training can enrich farmers' information access channels, obtain a large amount of information on pesticide use policies and markets (63), reverse farmers' disadvantage in information access, suppress the incompleteness of information dissemination among farmers, reduce information asymmetry, and improve farmers' risk cognition. In a word, participating in cooperatives training helps farmers to build a knowledge system of scientific use of pesticides and overcome information asymmetry to improve their risk cognition level, and finally promote scientific and rational use of pesticides.

### Mechanism test of the moderating effect of cooperatives training in risk cognition affecting overuse of pesticides

The previous empirical results show that there are differences in the effect of risk cognition on the overuse of pesticides between farmers who participate in cooperatives training and farmers who do not participate in cooperatives training. According to theoretical analysis, the difference in risk cognition caused by participating in cooperatives training may be the reason for this result. To verify this logic, this paper further analyzes the impact of cooperatives training on farmers' risk cognition (Table 6).

**Table 6:** Analysis of the impact of cooperative training on risk cognition.

**Variable name**	**Food safety risk cognition**		**Ecological environment risk cognition**		**Human health risk cognition**	
	**Coefficient**	**Standard error**	**Coefficient**	**Standard error**	**Coefficient**	**Standard error**
Cooperative training	0.366^**^	0.054	0.501^**^	0.062	0.437^*^	0.073
Control variables	Controlled		Controlled		Controlled	
Observed value	518		518		518	
*R* ^2^	0.107		0.121		0.116	

^*^, ^**^, respectively indicate that the significance level of 10, 5% was passed.

It can be seen that “whether to participate in cooperatives training” has a significant positive impact on risk cognition. It shows that compared with farmers who did not participate in cooperatives training, farmers who participated in cooperatives training were more likely to have a higher risk cognition of excessive pesticide use. Therefore, it can be explained that the effect of risk cognition on reducing excessive pesticide use is different among farmers who participate in cooperatives training or not, which is caused by the difference of risk cognition level between them.

### Robustness test

In order to verify the reliability of the above empirical results, this paper adopts the analytic hierarchy process to test the moderating effect of cooperatives training on the relationship between risk cognition and excessive pesticide use. Based on formula (1), the interaction items “cooperatives training × food safety risk cognition,” “cooperatives training × ecological environment risk cognition,” and “cooperatives training × human health risk cognition” are introduced for regression. It is not difficult to find that the interaction items “cooperatives training × food safety risk cognition,” “cooperatives training × ecological environment risk cognition,” and “cooperatives training × human health risk cognition” are all statistically significant at the 1% level, and the coefficients are positive. It shows that cooperatives training has a significant moderating effect on the risk cognition affecting farmers' excessive use of pesticides, which is consistent with the above regression results, indicating that the regression results are reliable (Table 7).

**Table 7:** Robustness test results.

**Variable name**	**Pesticide overuse**	
	**Coefficient**	**Standard error**
Cooperatives training × food safety risk cognition	0.686^***^	0.342
Cooperatives training × ecological environment risk cognition	0.406^***^	0.329
Cooperatives training × human health risk cognition	0.837^***^	0.402
Food safety risk cognition	−0.767^***^	0.240
Ecological environment risk cognition	−0.733^**^	0.243
Human health risk cognition	−1.143^***^	0.317
Cooperatives training	−5.424^***^	1.778
Control variables	Controlled	
Prob > chi^2^	0.000	
Pseudo *R*^2^	0.347	

^**^, ^***^, respectively indicate that the significance level of 5, and 1% was passed.

## Conclusions

Using the survey data of 518 vegetable growers in Shandong Province, China, this paper empirically tests the effect of risk cognition on farmers' pesticide overuse behavior and the moderating effect of cooperatives training on the effect of risk cognition on pesticide overuse behavior. The study found that: (1) risk cognition can significantly reduce the excessive use of pesticides. Food safety risk cognition, ecological environment risk cognition and human health risk cognition all have significant inhibitory effects on excessive use of pesticides, but the marginal effects of the three are different. The marginal effects of the three are in descending order: human health risk cognition, food safety risk cognition, and ecological environment risk cognition. (2) Cooperatives training has a positive moderating effect on the relationship between risk cognition and excessive use of pesticides, that is, the risk cognition of farmers participating in cooperatives training has a stronger inhibitory effect on their excessive use of pesticides than farmers who did not participate in cooperatives training. (3) The longer the farmers' education years, the larger the planting scale and the higher the detection frequency of government pesticide residues, the less the possibility of farmers' excessive use of pesticides.

According to the above conclusions, the following policy implications can be obtained: firstly, take risk cognition as an important policy reference for promoting pesticide reduction actions, and pay attention to the improvement of farmers' risk cognition level. On the one hand, the government should make full use of radio, television, newspapers, magazines, and other media to widely publicize the risks of excessive use of pesticides. The publicity work should focus on the loss of human health and food safety caused by excessive use of pesticides. On the other hand, universities, research institutes, and green agricultural enterprises are encouraged to enter villages for professional training and technical guidance, so as to improve vegetable farmers' cognitive level of pesticide application, establish correct pesticide application awareness and improve pesticide application efficiency. Secondly, strengthen the cultivation of agricultural cooperatives. Further publicize the law on agricultural cooperatives, support and encourage the development and expansion of cooperatives, and establish and improve the training and education system for cooperatives. The cooperatives provide information introduction, policy explanation, use consultation, skills training, personnel training, and other services for pesticide use, which can effectively improve farmers' risk cognition. On the basis of supporting cooperatives to carry out training and education, the government should further guide them to formulate supporting measures, encourage cooperatives to implement incentive and restraint mechanisms for farmers' safe use of pesticides, moderately manage and regulate farmers' production situation, and consolidate the training effect through supporting measures to encourage farmers' production practice. Thirdly, vigorously develop rural basic education to improve farmers' cultural level, speed up land circulation to promote land scale management, and strengthen the frequency and intensity of pesticide residue detection. These guarantee conditions are also effective points to promote farmers' scientific and rational use of pesticides. The government can also consider giving priority to those farmers who have received higher education, more training and larger farms to carry out pesticide use training, and then promote the improvement of all farmers' pesticide use knowledge through these farmers' transmission of pesticide use knowledge to other farmers.

This study also has some limitations: firstly, the study was conducted on randomly selected farmers planting vegetables in Shandong, China. The scale of the study object is relatively narrow. For other areas and other types of crops in China, whether the research conclusion can be applied and popularized needs further verification. In the future, the research area and crop types can be expanded, so as to obtain the general law of farmers' excessive use of pesticides. Secondly, we focus on discussion the impact of risk cognition and cooperatives training on farmers' excessive pesticide use behavior. However, farmers' production behavior is a complex decision-making problem, and farmers' excessive use of pesticides is bound to be affected by many factors. If more factors such as market and incentives can be brought into the analysis framework for discussion in the future, more valuable conclusions may be drawn.

## Data availability statement

The original contributions presented in the study are included in the article/supplementary material, further inquiries can be directed to the corresponding author/s.

## Ethics statement

The studies involving human participants were reviewed and approved by Shandong Normal University. The patients/participants provided their written informed consent to participate in this study.

## Author contributions

ZR: writing—original draft. HJ: reviewing and editing. Both authors contributed to the article and approved the submitted version.

## Conflict of interest

The authors declare that the research was conducted in the absence of any commercial or financial relationships that could be construed as a potential conflict of interest.

## Publisher's note

All claims expressed in this article are solely those of the authors and do not necessarily represent those of their affiliated organizations, or those of the publisher, the editors and the reviewers. Any product that may be evaluated in this article, or claim that may be made by its manufacturer, is not guaranteed or endorsed by the publisher.

## References

[1] Abhilash PC Singh N. Pesticide use and application: an Indian scenario. J Hazard Mater. (2009) 165:1–12. 10.1016/j.jhazmat.2008.10.061 19081675

[2] Liu Y Pan X Li J. A 1961–2010 record of fertilizer use, pesticide application and cereal yields: a review. Agron Sustain Dev. (2015) 35:83–93. 10.1007/s13593-014-0259-9

[3] Zhou JH Liu Q Liang Q. Cooperative membership, social capital, and chemical input use: evidence from China. Land Use Policy. (2018) 70:394–401. 10.1016/j.landusepol.2017.11.001

[4] Tripathy V Sharma KK Sharma K Gupta R Yadav R Singh G. Monitoring and dietary risk assessment of pesticide residues in brinjal, capsicum, tomato, and cucurbits grown in Northern and Western regions of India. J Food Composit Anal. (2022) 110:104543. 10.1016/j.jfca.2022.104543

[5] Zhou B Li X. The monitoring of chemical pesticides pollution on ecological environment by GIS. Environ Technol Innov. (2021) 23:101506. 10.1016/j.eti.2021.10150626789360

[6] Rani L Thapa K Kanojia N Sharma N Singh S Grewal AS. An extensive review on the consequences of chemical pesticides on human health and environment. J Clean Prod. (2021) 283:124657. 10.1016/j.jclepro.2020.12465732512419

[7] Mehmood Y Arshad M Mahmood N Kächele H Kong R. Occupational hazards, health costs, and pesticide handling practices among vegetable growers in Pakistan. Environ Res. (2021) 200:111340. 10.1016/j.envres.2021.111340 34043972

[8] Li ZJ. Prioritizing agricultural pesticides to protect human health: a multi-level strategy combining life cycle impact and risk assessments. Ecotoxicol Environ Saf. (2022) 242:113869. 10.1016/j.ecoenv.2022.113869 35835074

[9] Wang JH Tao JY Yang CC Chu M Lam H. A general framework incorporating knowledge, risk perception and practices to eliminate pesticide residues in food: a Structural Equation Modelling analysis based on survey data of 986 Chinese farmers. Food Control. (2017) 80143–150. 10.1016/j.foodcont.2017.05.003

[10] Shammi M Sultana A Hasan N Rahman MM Islam MS Bodrud-Doza M. Pesticide exposures towards health and environmental hazard in Bangladesh: a case study on farmers' perception. J Saudi Soc Agric Sci. (2020) 19:161–73. 10.1016/j.jssas.2018.08.005

[11] Zaller JG Kruse-Plaß M Schlechtriemen U Gruber E Peer M Nadeem I. Pesticides in ambient air, influenced by surrounding land use and weather, pose a potential threat to biodiversity and humans. Sci Tot Environ. (2022) 838:156012. 10.1016/j.scitotenv.2022.156012 35597361 PMC7614392

[12] Huang YZ Luo XF Tang L Yu WZ. The power of habit: does production experience lead to pesticide overuse?. Environ Sci Pollut Res. (2020) 27:25287–96. 10.1007/s11356-020-08961-4 32347493

[13] Sun SY Hu RF Zhang C. Pest control practices, information sources, and correct pesticide use: evidence from rice production in China. Ecol Indic. (2021) 129:107895. 10.1016/j.ecolind.2021.107895

[14] Grovermann C Schreinemachers P Riwthong S Berger T. ‘Smart’ policies to reduce pesticide use and avoid income trade-offs: an agent-based model applied to Thai agriculture. Ecol Econ. (2017) 132:91–103. 10.1016/j.ecolecon.2016.09.031

[15] Qin SL Lü XY. Do large-scale farmers use more pesticides? Empirical evidence from rice farmers in five Chinese provinces *J Integr Agric*. (2020) 19:590–9. 10.1016/S2095-3119(19)62864-9

[16] Yang M Zhao X Meng T. What are the driving factors of pesticide overuse in vegetable production? Evidence from Chinese farmers. China Agric Econ Rev. (2019) 11:672–87. 10.1108/CAER-08-2018-0170

[17] Jin JJ Wang WY He R Gong HZ. Pesticide use and risk perceptions among small-scale farmers in Anqiu county, China. Int J Environ Res Public Health. (2017) 14:29. 10.3390/ijerph14010029 28042850 PMC5295280

[18] Tang L Luo XF. Can agricultural insurance encourage farmers to apply biological pesticides? Evidence from rural China. Food Policy. (2021) 105:102174. 10.1016/j.foodpol.2021.102174

[19] Pan YX Ren YX Luning PA. Factors influencing Chinese farmers' proper pesticide application in agricultural products – A review. Food Control. (2021) 122:107788. 10.1016/j.foodcont.2020.107788

[20] Ubeda C Hornedo-Ortega R Cerezo AB Garcia-Parrilla MC Troncoso AM. Chemical hazards in grapes and wine, climate change and challenges to face. Food Chem. (2020) 314:126222. 10.1016/j.foodchem.2020.126222 31981884

[21] Monfared N Yazdanpanah M Tavakoli K. Why do they continue to use pesticides? The case of tomato growers in Boushehr Province in Southern Iran. J Agric Sci Technol. (2015) 17:577–88. Available online at: https://www.researchgate.net/publication/281728403

[22] Yazdanpanah M Moghadam MT Zobeidi T Turetta APD Eufemia L Sieber S. What factors contribute to conversion to organic farming? Consideration of the health belief model in relation to the uptake of organic farming by Iranian farmers. J Environ Plann Manage. (2021) 65:907–29. 10.1080/09640568.2021.1917348

[23] Yazdanpanah M Tajeri Moghadam M Javan F Deghanpour M Sieber S Falsafi P. How rationality, morality, and fear shape willingness to carry out organic crop cultivation: a case study of farmers in southwestern Iran. Environ Dev Sustain. (2022) 24:2145–63. 10.1007/s10668-021-01523-9

[24] Ajzen I. The theory of planned behavior. Organ Behav Hum Decis Process. (1991) 50:179–211. 10.1016/0749-5978(91)90020-T

[25] Slovic P. Perception of risk. Science. (1987) 236:280–5. 10.1126/science.3563507 3563507

[26] Bagheri A Emami N Damalas CA Allahyari MS. Farmers' knowledge, attitudes, and perceptions of pesticide use in apple farms of northern Iran: impact on safety behavior. Environ Sci Pollut Res. (2019) 26:9343–51. 10.1007/s11356-019-04330-y 30721432

[27] Pan D He MM Kong FB. Risk attitude, risk perception, and farmers' pesticide application behavior in China: a moderation and mediation model. J Clean Prod. (2020) 276:124241. 10.1016/j.jclepro.2020.124241

[28] Sharifzadeh MS Abdollahzadeh G Damalas CA Rezaei R Ahmadyousefi M. Determinants of pesticide safety behavior among Iranian rice farmers. Sci Tot Environ. (2019) 651:2953–60. 10.1016/j.scitotenv.2018.10.179 30463146

[29] Li MY Wang JJ Zhao PJ Chen K Wu LB. Factors affecting the willingness of agricultural green production from the perspective of farmers' perceptions. Sci Tot Environ. (2020) 738:140289. 10.1016/j.scitotenv.2020.140289 32806378

[30] Yu LY Huang W. Non-economic societal impact or economic revenue? A performance and efficiency analysis of farmer cooperatives in China. J Rural Stud. (2020) 80:123–34. 10.1016/j.jrurstud.2020.08.01017192115

[31] Deng HS Huang JK Xu ZG Rozelle S. Policy support and emerging farmer professional cooperatives in rural China. China Econ Rev. (2010) 21:495–507. 10.1016/j.chieco.2010.04.009

[32] Hao JH Bijman J Gardebroek C Heerink N Heijman W Huo XX. Cooperative membership and farmers' choice of marketing channels – Evidence from apple farmers in Shaanxi and Shandong Provinces, China. Food Policy. (2018) 74:53–64. 10.1016/j.foodpol.2017.11.004

[33] Wu Q Zhou J. Need for cognitive closure, information acquisition and adoption of green prevention and control technology. Ecol Chem Eng S. (2021) 28:129–43. 10.2478/eces-2021-0011

[34] Goeb J Lupi F. Showing pesticides' true colors: the effects of a farmer-to-farmer training program on pesticide knowledge. J Environ Manage. (2021) 279:111821. 10.1016/j.jenvman.2020.111821 33340964

[35] Sarkar A Wang HY Rahman A Qian L Memon WH. Evaluating the roles of the farmer's cooperative for fostering environmentally friendly production technologies-a case of kiwi-fruit farmers in Meixian, China. J Environ Manage. (2022) 301:113858. 10.1016/j.jenvman.2021.113858 34607139

[36] Mgendi BG Mao SP Qiao FB. Does agricultural training and demonstration matter in technology adoption? The empirical evidence from small rice farmers in Tanzania. Technol Soc. (2022) 70:102024. 10.1016/j.techsoc.2022.102024

[37] Sun SY Zhang C Hu RF. Determinants and overuse of pesticides in grain production: a comparison of rice, maize and wheat in China. China Agric Econ Rev. (2020) 12:367–79. 10.1108/CAER-07-2018-0152

[38] Mu HY Zhang JC Yang XM Wang K Xu W Zhang HY. Pesticide screening and health risk assessment of residential dust in a rural region of the North China Plain. Chemosphere. (2022) 303:135115. 10.1016/j.chemosphere.2022.135115 35636607

[39] Wang R Yang Y Deng Y Hu DY Lu P. Multiresidue analysis and dietary risk assessment of pesticides in eight minor vegetables from Guizhou, China. Food Chem. (2022) 380:131863. 10.1016/j.foodchem.2021.131863 34996635

[40] Krejcie RV Morgan DW. Determining sample size for research activities. Educ Psychol Meas. (1970) 30:607–10. 10.1177/001316447003000308

[41] Lai WY. Pesticide use and health outcomes: evidence from agricultural water pollution in China. J Environ Econ Manage. (2017) 86:93–120. 10.1016/j.jeem.2017.05.00635063521

[42] Li M Yan XBB Guo YQ Ji H. Impact of risk awareness and agriculture cooperatives' service on farmers' safe production behaviour: evidences from Shaanxi Province. J Clean Prod. (2021) 312:127724. 10.1016/j.jclepro.2021.127724

[43] Liu Y Ruiz-Menjivar J Zhang L Zhang JB Swisher ME. Technical training and rice farmers' adoption of low-carbon management practices: the case of soil testing and formulated fertilization technologies in Hubei, China. J Clean Prod. (2019) 226:454–62. 10.1016/j.jclepro.2019.04.026

[44] Zhou L Zhang F Zhou S Turvey CG. The peer effect of training on farmers' pesticides application: a spatial econometric approach. China Agric Econ Rev. (2020) 12:481–505. 10.1108/CAER-01-2019-0003

[45] Young JC Calla S Lécuyer L Skrimizea E. Understanding the social enablers and disablers of pesticide reduction and agricultural transformation. J Rural Stud. (2022) 95:67–76. 10.1016/j.jrurstud.2022.07.023

[46] Jallow MFA Awadh DG Albaho MS Devi VY Thomas BM. Pesticide risk behaviors and factors influencing pesticide use among farmers in Kuwait. Sci Tot Environ. (2017) 574:490–8. 10.1016/j.scitotenv.2016.09.085 27644027

[47] James LR Brett JM. Mediators, moderators, and tests for mediation. J Appl Psychol. (1984) 69:307–21. 10.1037/0021-9010.69.2.307

[48] Baron RM Kenny DA. The moderator-mediator variable distinction in social psychological research: conceptual, strategic, and statistical considerations. J Pers Soc Psychol. (1986) 51:1173–82. 10.1037/0022-3514.51.6.1173 3806354

[49] Ahmad D Afzal M Rauf A. Analysis of wheat farmers' risk perceptions and attitudes: evidence from Punjab, Pakistan. Nat Hazards. (2019) 95:845–61. 10.1007/s11069-018-3523-5

[50] Asravor RK. Farmers' risk preference and the adoption of risk management strategies in Northern Ghana. J Environ Plann Manage. (2019) 62:881–900. 10.1080/09640568.2018.1452724

[51] Kuruppu N Liverman D. Mental preparation for climate adaptation: the role of cognition and culture in enhancing adaptive capacity of water management in Kiribati. Glob Environ Change. (2011) 21:657–69. 10.1016/j.gloenvcha.2010.12.002

[52] Mohan S. Risk aversion and certification: evidence from the Nepali tea fields. World Dev. (2020) 129:104903. 10.1016/j.worlddev.2020.104903

[53] Knowler D Bradshaw B. Farmers' adoption of conservation agriculture: a review and synthesis of recent research. Food Policy. (2007) 32:25–48. 10.1016/j.foodpol.2006.01.003

[54] Chen X Zeng D Xu Y Fan X. Perceptions, risk attitude and organic fertilizer investment: evidence from rice and banana farmers in Guangxi, China. Sustainability. (2018) 10:3715. 10.3390/su10103715

[55] Cao H Zhu X Heijman W Zhao K. The impact of land transfer and farmers' knowledge of farmland protection policy on pro-environmental agricultural practices: the case of straw return to fields in Ningxia, China. J Clean Prod. (2020) 277:123701. 10.1016/j.jclepro.2020.123701

[56] Wang HY Wang XL Sarkar A Zhang FH. How capital endowment and ecological cognition affect environment-friendly technology adoption: a case of apple farmers of Shandong Province, China. Int J Environ Res Public Health. (2021) 18:7571. 10.3390/ijerph18147571 34300022 PMC8305192

[57] Sharafi K Pirsaheb M Maleki S Arfaeinia H Karimyan K Moradi M. Knowledge, attitude and practices of farmers about pesticide use, risks, and wastes; a cross-sectional study (Kermanshah, Iran). Sci Tot Environ. (2018) 645:509–17. 10.1016/j.scitotenv.2018.07.132 30029126

[58] Zhao L Wang C Gu H Yue C. Market incentive, government regulation and the behavior of pesticide application of vegetable farmers in China. Food Cont. (2018) 85:308–17. 10.1016/j.foodcont.2017.09.01636159273

[59] Zhu P Zheng J Zhang M Liu Y. Effect of joining cooperatives on grain grower's behavior of green production technology adoption and driving mechanism. J Arid Land Resour Environ. (2022) 36:67–75. 10.13448/j.cnki.jalre.2022.253

[60] Fernando S Garnevska E Ramilan T Shadbolt N. Organisational attributes of cooperatives and farmer companies. J Co-oper Organ Manage. (2021) 9:100132. 10.1016/j.jcom.2021.100132

[61] Ji C Chen Q Trienekens J Wang HT. Determinants of cooperative pig farmers' safe production behaviour in China — Evidences from perspective of cooperatives' services. J Integr Agric. (2018) 17:2345–55. 10.1016/S2095-3119(18)62058-1

[62] Waichman AV Eve E Nina NCDS. Do farmers understand the information displayed on pesticide product labels? A key question to reduce pesticides exposure and risk of poisoning in the Brazilian Amazon. Crop Protect. (2007) 26:576–83. 10.1016/j.cropro.2006.05.011

[63] Jin SQ Bluemling B Mol APJ. Information, trust and pesticide overuse: interactions between retailers and cotton farmers in China. J Life Sci. (2015) 72–3:23–32. 10.1016/j.njas.2014.10.003

